# Accelerated onset of CNS prion disease in mice co-infected with a gastrointestinal helminth pathogen during the preclinical phase

**DOI:** 10.1038/s41598-020-61483-4

**Published:** 2020-03-12

**Authors:** David S. Donaldson, Barry M. Bradford, Kathryn J. Else, Neil A. Mabbott

**Affiliations:** 1 The Roslin Institute & Royal (Dick) School of Veterinary Sciences, University of Edinburgh, Easter Bush, EH25 9RG Midlothian, United Kingdom; 2 Lydia Becker Institute of Immunology and Inflammation, School of Biological Sciences, Faculty of Biology, Medicine and Health, University of Manchester, Manchester, M13 9PL United Kingdom

**Keywords:** Neuroimmunology, Encephalopathy, Infection, Parasite host response, Astrocyte

## Abstract

Prion infections in the central nervous system (CNS) can cause extensive neurodegeneration. Systemic inflammation can affect the progression of some neurodegenerative disorders. Therefore, we used the gastrointestinal helminth pathogen *Trichuris muris* to test the hypothesis that a chronic systemic inflammatory response to a gastrointestinal infection would similarly affect CNS prion disease pathogenesis. Mice were injected with prions directly into the CNS and subsequently orally co-infected with *T. muris* before the onset of clinical signs. We show that co-infection with a low dose of *T. muris* that leads to the development of a chronic T helper cell type 1-polarized systemic immune response accelerated the onset of clinical prion disease. In contrast, co-infection with a high dose of *T. muris* that induces a T helper cell type 2-polarized immune response did not affect prion disease pathogenesis. The reduced survival times in mice co-infected with a low dose of *T. muris* on d 105 after CNS prion infection coincided with enhanced astrocyte activation in the brain during the preclinical phase. These data aid our understanding of how systemic inflammation may augment the progression of neurodegeneration in the CNS.

## Introduction

Infections with prions cause chronic neurodegenerative diseases to which there are no effective treatments. During prion disease PrP^Sc^ (abnormally folded isoforms of the host’s cellular prion protein, PrP^C^) accumulates in affected tissues. The build-up of PrP^Sc^ within the brain during prion disease ultimately leads to the development of spongiform pathology (vacuolation) and neurodegeneration, and coincides with extensive astrocytic and microglial activation in targeted regions^1^. Infectious prion particles appear to be comprised almost entirely of PrP^Sc^, suggesting that prions are infectious proteins^2,3^. Understanding the factors that affect the risk of developing clinical prion disease is important for developing treatments and managing disease risk.

Pathogen infection and systemic inflammation can affect the progression of some neurodegenerative disorders. For example, systemic inflammation has been associated with increased cognitive decline in Alzheimer’s disease patients^4,5^, and enhanced neuropathology in a mouse tauopathy model^6^. A prospective cohort study has similarly proposed that gastrointestinal infections increase the risk of Parkinson’s disease^7^. A systemic inflammatory response to infection with a gastrointestinal helminth pathogen can also enhance brain injury in experimental stroke^8^. However, in some circumstances the effects of certain helminth infections on the host could have a beneficial impact, such as dampening autoimmune responses and disease severity in multiple sclerosis patients^9,10^. A negative correlation between infection with *Helicobater pylori* seropositivity and susceptibility to multiple sclerosis has also been described^11^.

The commensal gut microbiota may also play a role in the progression of some neurodegenerative disorders. For example, gut microbiota induced alterations in the induction of peripheral immune tolerance can modulate the development of experimental autoimmune encephalitis in mice^12^. Furthermore, dysbiosis of the commensal gut microbiota is commonly observed in multiple sclerosis patients and may play a pathological role in the initiation and progression of this disease^13^. Effects of dysbiosis on the production of certain commensal-derived metabolites may similarly modulate the neurodegenerative disorder amyotrophic lateral sclerosis^14^.

The microglia are the mononuclear phagocytes of the brain and play important roles in maintaining neuronal homeostasis, removing dying cells and protecting against pathogen infection. These cells can play a host-protective role during CNS prion disease by phagocytosing and destroying the prions^15,16^. However, modifications to microglial activation status can affect the rate of the development of the neuropathology^17,18^. For example, systemic (intraperitoneal) exposure to bacterial LPS induces a pro-inflammatory microglial phenotype^19^ that exacerbates the neuropathology and accelerates the onset of the clinical signs of prion disease^20^.

The astrocytes in the brain similarly provide homeostatic support to neurons in the steady state, but undergo reactive astrocytosis following brain injury and during neurodegenerative diseases. Reactive astrocytes can be divided into two main functional subclasses^21^. The A1 subclass of reactive astrocytes exhibit neurotoxic features, whereas the A2 astrocytes express neurotrophic factors and are considered to be neuroprotective^21^. Reactive astrocytes are also induced in the brain during prion disease, but it is uncertain whether they provide protection from, or contribute to the neurodegeneration. A study using transgenic mice in which PrP^C^ was exclusively expressed in astrocytes has shown that these cells can also support prion replication and this can lead to neuropathology^22^. A separate *in vitro* study proposed that reactive astrocytes recruit microglia into brain regions infected with prions^23^. Peripheral LPS exposure can also stimulate the development of neurotoxic reactive A1 astrocytes^21^. This raises the possibility that systemic inflammatory mediators may similarly manipulate the activation phenotype of astrocytes and as a consequence modulate CNS prion disease progression.

*Trichuris muris* is an extensively characterised natural mouse gastrointestinal helminth pathogen. After oral exposure the *T. muris* infection is entirely restricted to the large intestine where the parasite burrows into the epithelium^24^. The characteristics of the host’s immune response to *T. muris* infection are influenced by the magnitude of the infectious dose used. Oral infection of C57Bl/6J mice with a high dose of infective *T. muris* eggs (~200) induces a protective CD4+ T helper cell type 2 (Th2)-polarized immune response. Conversely, low dose *T. muris* infection (~20 infective eggs) induces a chronic parasite-specific Th1-polarized response that is non-protective^25–27^. A previous study has shown that a systemic *T. muris*-specific Th1-polarized immune response can exacerbate the neuropathology in a murine model of stroke^8^. We therefore used *T. muris* to test the hypothesis that a systemic inflammatory response to co-infection with a gastrointestinal helminth pathogen would similarly affect CNS prion disease pathogenesis. We show that a low dose of *T. muris* infection accelerates CNS prion disease and this is associated with earlier astrocyte activation. Data from this study will aid our understanding of the important factors that can affect the risk of developing clinical prion disease.

## Results

### Co-infection with a low dose of *T. muris* accelerates the onset of CNS prion disease

**Figure 1:**
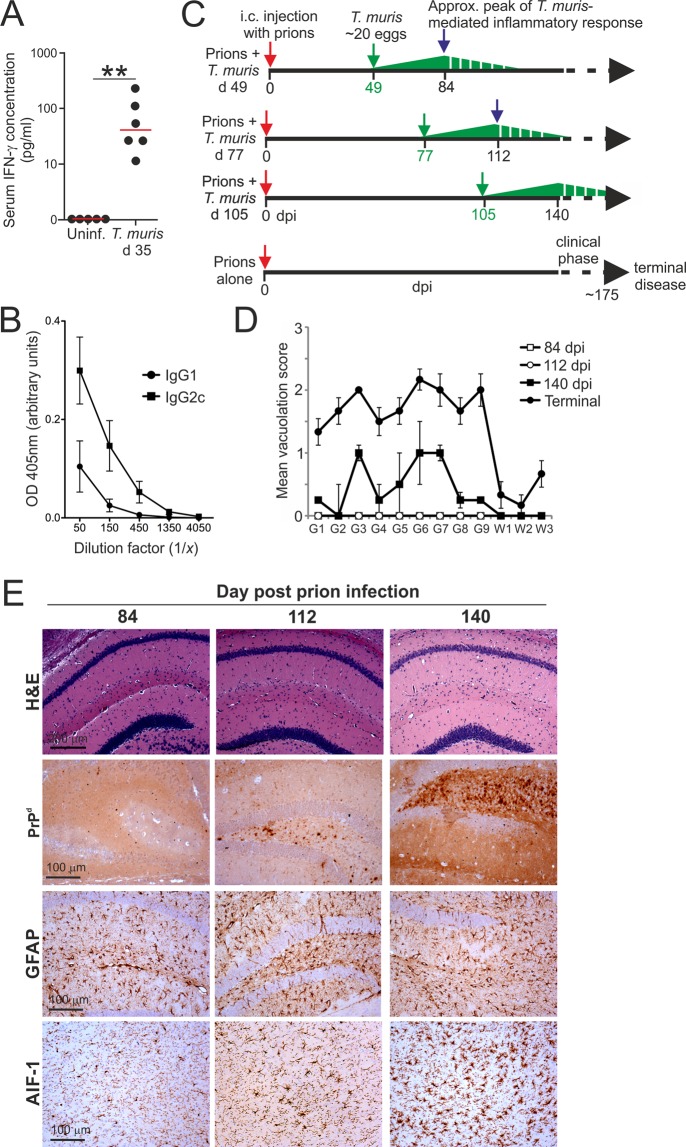
Experimental Design. (**A,B**) Mice were orally infected with a low dose of *T. muris* infective eggs (~20/mouse) and serum collected 35 d later. (**A**) Comparison of IFN-γ concentrations in the serum of *T. muris* infected mice and uninfected (uninf.) control mice. Each point represents data from an individual mouse (*n* = 5–6 mice/group). **P < 0.01, Student’s *t*-test. Horizontal bars, median. (**B**) Analysis of *T. mu*ris E/S antigen-specific IgG1 (closed circles) and IgG2c (closed squares) levels in the sera of *T. muris* infected mice. Each point represents the mean OD 405 nm ± SEM, *n* = 3 mice/group. (**C**) Experiment design. Cartoon shows the relative timings of the individual oral *T. muris* infections in relation to the ongoing prion infection in the CNS. Dpi, d post IC prion infection; green arrows, d on which mice were orally infected with a single low dose of *T. muris*. The green triangles represent the relative magnitude of the *T. muris*-specific immune response, with the peak response occurring by ~35 d and gradually declining afterwards. (**D,E**) Mice were injected IC with ME7 scrapie prions directly into the CNS and 84, 112 and 140 d later brains were collected and the neuropathology compared by histopathology. (**D**) The severity of the spongiform pathology (vacuolation) within each brain was scored on a scale of 1–5 in nine grey matter and three white matter areas: G1, dorsal medulla; G2, cerebellar cortex; G3, superior colliculus; G4, hypothalamus; G5, thalamus; G6, hippocampus; G7, septum; G8, retrosplenial and adjacent motor cortex; G9, cingulate and adjacent motor cortex; W1, inferior and middle cerebellar peduncles; W2, decussation of superior cerebellar peduncles; and W3, cerebellar peduncles. Each point represents the mean vacuolation score ± SEM, *n* = 4 mice/group. Scores from mice with terminal ME7 prion disease are included for comparison. (**E**) Histopathological comparison of the spongiform pathology (H&E, upper row), PrP^d^ accumulation (brown, second row), reactive astrocytes expressing GFAP (brown, third row) and active microglia expressing AIF-1 (brown, bottom row) in the brains of mice at times indicated after IC injection with prions. Haematoxylin was used as a nuclear counterstain (blue). *n* = 4 mice/group.

As anticipated, oral infection of C57BL/6J mice with a low dose of ~20 *T. muris* infective eggs stimulated the production of elevated levels of IFN-γ (Fig. 1A) and IFN-γ-induced parasite-specific IgG2c in the serum (Fig. 1B)^8,25,26^. These data were consistent with the induction of a chronic parasite–specific Th1-polarised antibody response to low dose *T. muris* infection, that is prevented when IFN-γ is depleted^26^. To determine whether the host’s response to low dose *T. muris* infection might influence the pathogenesis of an ongoing prion infection in the brain, groups of C57BL/6J mice were injected with ME7 scrapie prions directly into the CNS by intracerebral (IC) injection. The IC route of prion infection was used here as the timing of the development of the neuropathology has been extensively characterised^28^. This also allowed compartmentalisation of the two pathogen infections to distinct niches: *T. muris* to the large intestine; prions to the CNS. Subsequently, individual groups of these mice were orally co-infected with a single low dose of ~20 *T. muris* infective eggs on one of the following days: d 49, d 77 or d 105 post IC injection with prions (Fig. 1C). The parasite infections were timed such that the peak of the parasite-driven inflammatory response typically encountered at ~35 d post-*T. muris* infection^8,25,26^ would coincide with the following pre-clinical phases of the on-going prion infection in the brain (Fig. 1C–E):

- 84 d after IC prion infection, prior to the onset of detectable neuropathology within the brain (Fig. 1D, open squares; Fig. 1E, left-hand panels).
- 112 d after IC prion infection, around the onset of detectable neuropathology within the brain (Fig. 1D, open circles; Fig. 1E, middle panels).
- 140 d after IC prion infection, when signs of neuropathology (spongiform pathology, PrP^d^ accumulation and activation of astrocytes and microglia) are detectable within the brain but just before the onset of observable clinical signs of disease (Fig. 1D, closed squares; Fig. 1E, right-hand panels).

**Figure 2:**
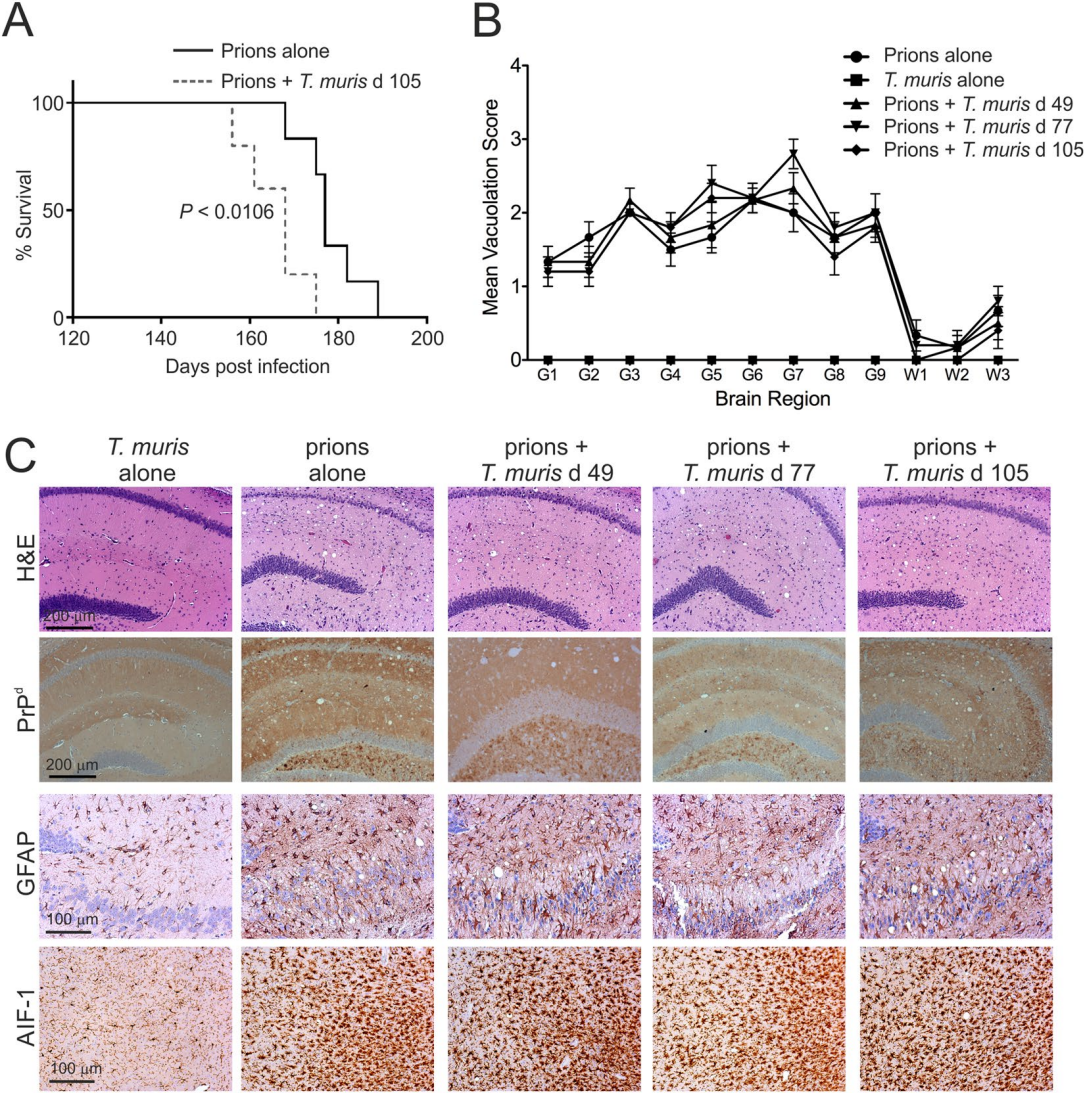
Co-infection with a low dose of *T. muris* does not affect the development of neuropathology at the terminal stage of prion disease. Mice were first injected with ME7 scrapie prions directly into the CNS by IC injection. Then, at the times indicated after prion infection the mice were orally co-infected with a low dose of *T. muris* infective eggs (~20/mouse; 5–6 mice/group). (**A**) Co-infection with a low dose of *T. muris* at 105 d after prion infection significantly reduced the clinical prion disease survival time when compared to mice infected with prions alone (*P* < 0.0106, Log-rank [Mantel-Cox] test). (**B**) The severity of the spongiform pathology (vacuolation) within each brain was scored on a scale of 1–5 in nine grey matter and three white matter areas as described in the legend to Fig. 1. Each point represents the mean vacuolation score ± SEM, *n* = 5–6 mice/group. (**C**) Brains were collected from all mice at the terminal stage of prion disease and the neuropathology compared. High levels of spongiform pathology (H&E, upper row), heavy accumulations of PrP^d^ (brown, second row), reactive astrocytes expressing GFAP (brown, third row) and active microglia expressing AIF-1 (brown, bottom row) were detected in the brains of all the terminally-affected mice. Haematoxylin was used as a nuclear counterstain (blue). *n* = 5–6 mice/group. None of the histopathological signs of prion disease were detected in the brains of mice infected with a low dose of *T. muris* alone.

As anticipated, the control mice injected with prions alone developed clinical prion disease with a mean survival time of 178 ± 3 d (median = 177 d; Table 1). Mice co-infected with a low dose of *T. muris* at 49 or 77 d after prion infection also succumbed to clinical prion disease with similar survival times to those infected with prions alone (Table 1). However, a significant reduction in the mean survival time of approximately 12 d was observed when the mice were co-infected with a low dose of *T. muris* at 105 d after prion infection (Table 1, mean = 166 ± 3 d, median = 168 d, *P* < 0.019; Fig. 2A, survival curve, prions alone vs. co-infection with a low dose of *T. muris* at 105 d after prion infection, *P* < 0.0106, Log-rank [Mantel-Cox] test). The reduced survival times in these co-infected mice were not associated with a reduced duration of the clinical phase of prion disease (Table 1). Instead, co-infection with *T. muris* at 105 d after prion infection significantly reduced the mean onset of detectable clinical signs by approximately 9 d (low dose *T. muris* at 105 d, mean clinical onset = 143 ± 2 d; prions alone, mean clinical onset = 152 ± 3 d; *P* < 0.035; Table 1).

**Table 1:** Effect of co-infection with *T. muris* on CNS prion disease.

Mouse model	*T. muris* dose	Timing of oral *T. muris* infection^a^ (days)	Mean onset of clinical prion disease (days)^b^	Mean duration of clinical prion disease (days)^c^	Mean survival times (days)^d^	Median survival times (days)	Clinical disease^e^
Prions alone		none	152 ± 3	26 ± 2	178 ± 3	177	6/6
Prions + *T. muris*	Low	49	152 ± 1 NS	25 ± 2 NS	177 ± 3 NS	179	6/6
Prions + *T. muris*	Low	77	154 ± 4 NS	23 ± 5 NS	177 ± 4 NS	180	6/6
Prions + *T. muris*	Low	105	143 ± 2 *P* < 0.035^f^	22 ± 2 NS	166 ± 3 *P* < 0.019	168	5/5
Prions + *T. muris*	High	63	157 ± 3 NS	27 ± 3 NS	183 ± 3 NS	183	6/6
Prions + *T. muris*	High	91	161 ± 4 NS	19 ± 3 NS	180 ± 3 NS	177	6/6
Prions + *T. muris*	High	119	150 ± 3 NS	29 ± 2 NS	177 ± 4 NS	177	6/6

^a^All mice were injected IC with prions on the same day. On the days indicated after prion injection the mice were subsequently orally-infected with either a low dose (~20) or high dose (~200) of *T. muris* infective eggs.

^b^Mean d ± SEM after IC injection with prions on which the first clinical signs of disease were observed.

^c^Mean duration ± SEM from first clinical signs of prion disease to development of terminal signs of clinical disease.

^d^Mean duration ± SEM from IC injection with prions to development of terminal signs of clinical disease.

^e^Incidence = no. animals displaying clinical signs of prion disease/no. animals tested.

^f^Statistical differences when compared to mice injected with prions alone were compared by one-way ANOVA and Student’s *t*-test. NS, not significant.

Co-infection with a low dose of *T. muris* did not affect the magnitude of the spongiform pathology in the brain at the terminal stage of prion disease, when compared to mice infected with prions alone (Fig. 2B,C upper row). Heavy deposits of prion disease-specific PrP (PrP^d^) were also detected in the brains of mice from each group, and co-infection with a low dose of *T. muris* did not influence their abundance (Fig. 2C). The brains of mice with terminal prion disease also display increased astrocyte and microglial activation. Here, glial fibrillary acidic protein (GFAP) immunostaining was used to detect reactive astrocytes, and allograft inflammatory factor-1 (AIF-1, also known as Iba1) immunostaining was used to detect microglia. Immunohistochemical (IHC) analysis showed extensive astrogliosis and microgliosis in the brains of mice infected with prions alone and in those that were co-infected with a low dose *T. muris* at 105 d after prion infection (Fig. 2C). None of these neuropathological signs were detected in the brains of mice infected with *T. muris* alone as a control (Fig. 2B,C). These data show that co-infection with a low dose of *T. muris* did not affect the magnitude of the neuropathology in the brain (spongiform pathology, PrP^d^ accumulation, astrocytosis and microgliosis) in clinically-affected mice.

### Co-infection with a high dose of *T. muris* does not influence CNS prion disease pathogenesis

**Figure 3:**
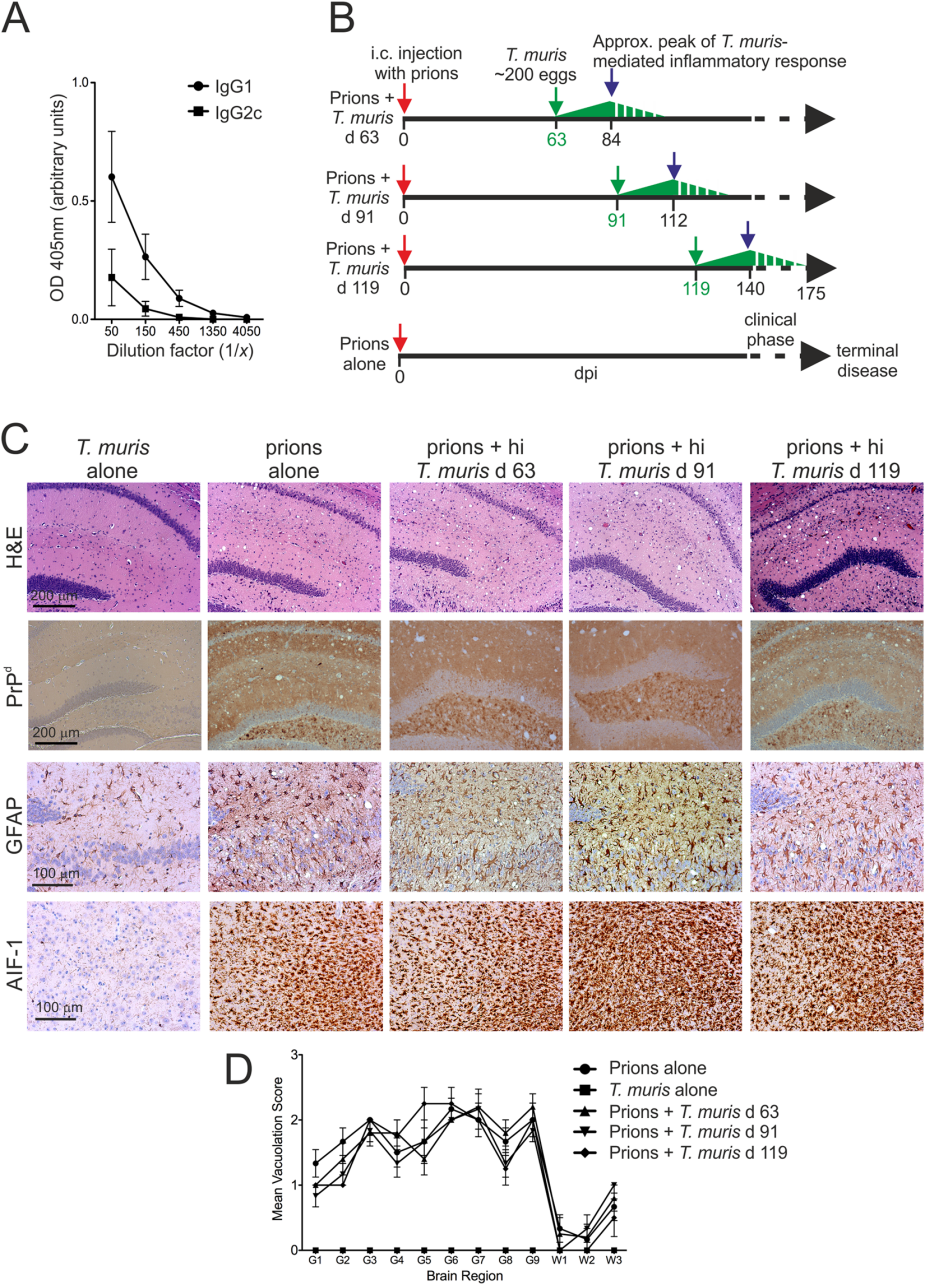
Co-infection with a high dose of *T. muris* does not affect the development of neuropathology at the terminal stage of prion disease. (**A**) Analysis of *T. mu*ris E/S antigen-specific IgG1 (closed circles) and IgG2c (closed squares) levels in the sera of mice 21 d after high dose *T. muris* infection (~200 infective eggs). Each point represents the mean OD 405 nm ± SEM, *n* = 3 mice/group. (**B**) Experiment design. Cartoon shows the relative timings of the oral *T. muris* infections in relation to the ongoing prion infection in the CNS. Dpi, d post IC prion infection; green arrows, d on which mice were orally infected with a single high dose of *T. muris* infective eggs. The green triangles represent the relative magnitude of the *T. muris*-specific immune response, with the peak response occurring by ~21 d and gradually declining afterwards. (**C**) Mice were injected with ME7 scrapie prions directly into the CNS by IC injection and subsequently orally infected with a high dose of *T. muris* infective eggs at the times indicated (*n* = 6 mice/group). Brains were collected from all mice with terminal prion disease and the neuropathology compared. High levels of spongiform pathology (H&E, upper row), heavy accumulations of PrP^d^ (brown, second row), reactive astrocytes (GFAP+ cells, brown, third row) and active microglia (AIF-1+ cells, brown, bottom row) were detected in the brains of all the terminally-affected mice. Haematoxylin was used as a nuclear counterstain (blue). None of the histopathological signs of prion disease were detected in the brains of mice infected with a high dose of *T. muris* alone. (**D**) The severity of the spongiform pathology (vacuolation) within each brain was scored on a scale of 1–5 in nine grey matter and three white matter areas as described in the legend to Fig. 1. Each point represents the mean vacuolation score ± SEM, *n* = 6 mice/group.

Oral infection of C57BL/6J mice with a high dose of ~200 infective *T. muris* eggs induces the development of an acute, protective, Th2-polarised immune response that is associated with high levels of parasite-specific IgG1 in the serum (Fig. 3A)^8,25–27^. We next determined whether the effect of *T. muris* on CNS prion disease was specific to co-infection with helminths or the nature of the immune response to the helminth infection. Mice were injected IC with prions and subsequently orally co-infected with a single high dose of ~200 infective *T. muris* eggs on one of three distinct intervals after the prion infection (Fig. 3B). As above, the *T. muris* infections were timed such that the peak Th2-polarized inflammatory response (~21 d post-*T. muris* infection) would occur before or during the development of neuropathology within the brain (Fig. 3B).

All mice co-infected with a high dose of *T. muris* developed clinical prion disease with survival times equivalent to those infected with prions-alone (Table 1). Co-infection with a high dose of *T. muris* also did not alter the timing of the onset or the duration of the clinical phase of prion disease (Table 1), or the severity of the neuropathology in the brain at the terminal stage of disease (Fig. 3C,D).

These data show that the effects of co-infection with *T. muris* on CNS prion disease were specific to infection with a low dose of infective eggs. This raised the hypothesis that factors specific to the development of a chronic parasite–specific Th1-polarised immune response may have accelerated the development of the neuropathology during the pre-clinical phase.

### Effect of *T. muris* infection on systemic CCL5 synthesis

**Figure 4:**
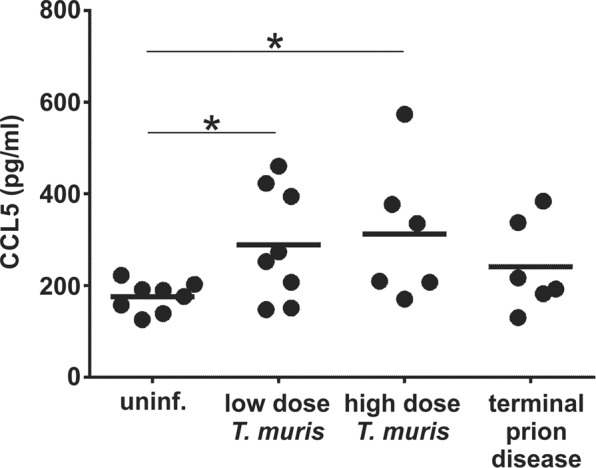
Serum CCL5 levels in *T. muris* infected mice and mice with terminal prion disease. Serum was isolated from peripheral blood collected from uninfected mice (uninf.), 21 d after low dose of *T. muris* infection (~20 infective eggs/mouse), 35 d after high dose of *T. muris* infection (~200 infective eggs) and at the terminal stage of prion disease. Each point represents data from an individual mouse, *n* = 6–8 mice/group. Horizontal line, median. *P < 0.05, Students *t*-test.

An independent study^8^ has suggested that a chronic Th1-polarized inflammatory response after low dose *T. muris* infection augments ischemic brain injury in mice via systemic production of the chemokine CCL5. Our analysis confirmed that CCL5 was significantly elevated in the sera of mice infected with a low dose of *T. muris*, but equally high levels were detected in mice infected with a high dose of *T. muris* (Fig. 4). Furthermore, serum CCL5 levels were also elevated in some of the mice with clinical prion disease (Fig. 4). These data suggested that factors other than systemic CCL5 expression were likely to be responsible for the accelerated onset of CNS prion disease pathogenesis in mice co-infected with a low-dose of *T. muris*.

### Earlier onset of astrocyte activation in mice co-infected with a low dose of *T. muris* during the pre-clinical phase of prion disease

**Figure 5:**
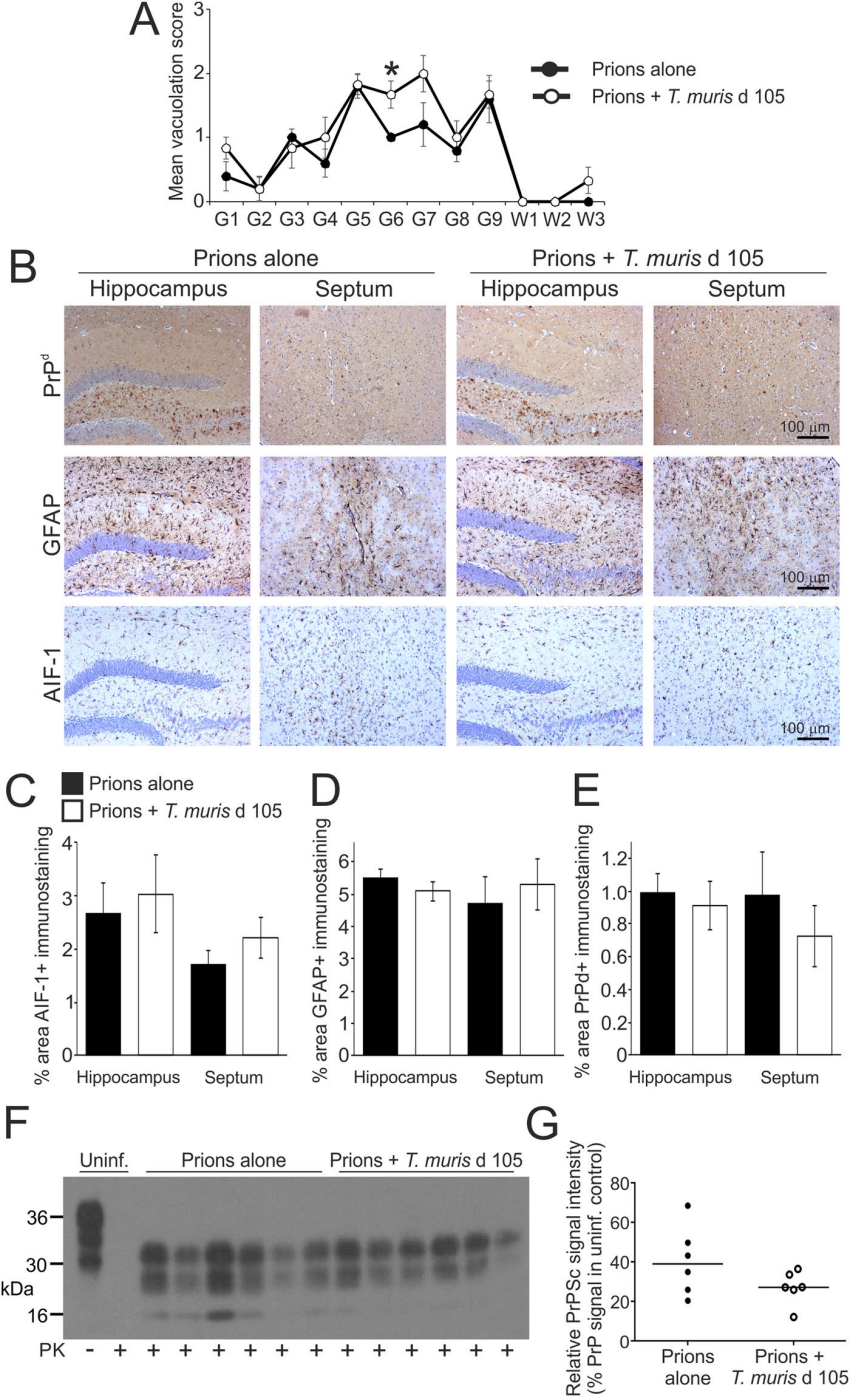
Effect of co-infection with a low dose of *T. muris* on the development of neuropathology during the pre-clinical phase of prion disease. Mice were first injected with ME7 scrapie prions directly into the CNS by IC injection and 105 d later they were orally co-infected with a low dose of *T. muris* infective eggs (~20/mouse). Brains were collected at 140 d after prion infection during the pre-clinical phase of disease and the neuropathology compared. (**A**) The severity and distribution of the spongiform pathology (vacuolation) within each brain was scored on a scale of 1–5 in nine grey matter and three white matter areas as described in the legend to Fig. 1. Each point represents the mean vacuolation score ± SEM, *n* = 5–6 mice/group. *P < 0.05, Students *t*-test. (**B**) Immunohistochemical analysis of PrP^d^ accumulation (brown, upper row), reactive astrocytes (GFAP+ cells, brown, middle row) and active microglia (AIF-1+ cells, brown, bottom row) in the hippocampus and septum of mice from each group. Haematoxylin was used as a nuclear counterstain (blue). (**C–E**) Morphometric analysis suggested that the area of the (**C**) AIF-1+, (**D**) GFAP+ and (**E**) PrP^d^+ immunostaining in the hippocampus and septum was similar in the brains of mice co-infected with *T. muris* (open bars), when compared to mice infected with prions alone (closed bars). Data represent mean ± SEM, *n* = 5–6 mice/group, not significantly different to prions alone, Mann-Whitney *U* test (**C**) and Student’s *t*-test (**D,E**). (**F**) Immunoblot shows similar levels of proteinase (PK) resistant PrP^Sc^ in brains of mice from each treatment group collected at 140 d after prion infection. Uninf., uninfected (control) mice. Three bands are characteristically detected after PK treatment representing the un-glycosylated, mono-glycosylated, and di-glycosylated isomers of PrP^Sc^. (**G**) Densitometric comparison of PrP^Sc^ detected by immunoblot in brains of mice infected with prions alone or co-infected with prions or *T. muris*. Data are presented as relative % PrP signal in uninfected control. *n* = 6 mice/group, not significantly different, Student’s *t*-test.

We next determined whether the earlier onset of clinical signs in mice co-infected with a low dose of *T. muris* at 105 d post prion infection coincided with enhanced neuropathology during the pre-clinical phase. Mice were injected IC with prions, co-infected with a low dose of *T. muris* 105 d later, and brains analysed at 140 d post prion infection. Age-matched uninfected mice and those infected with prions alone or *T. muris* alone were used as controls. At 140 d post prion infection, a modest increase in the magnitude of the spongiform pathology was observed in the hippocampus and septum regions (G6 and G7 regions, respectively) of the brains of mice co-infected with *T. muris* when compared to mice infected with prions alone (Fig. 5A). However, comparison of the magnitude of the AIF-1+ and GFAP+ immunostaining suggested a similar abundance of reactive microglia (Fig. 5B,C) and astrocytes (Fig. 5B,D) respectively, in these regions at this time after prion infection. Similar levels of PrP^d^ (IHC; Fig. 5B,E) and PrP^Sc^ (immunoblot; Fig. 5F,G) accumulation were also detected in the brains of mice infected with prions alone and those co-infected with prions and *T. muris*.

**Figure 6:**
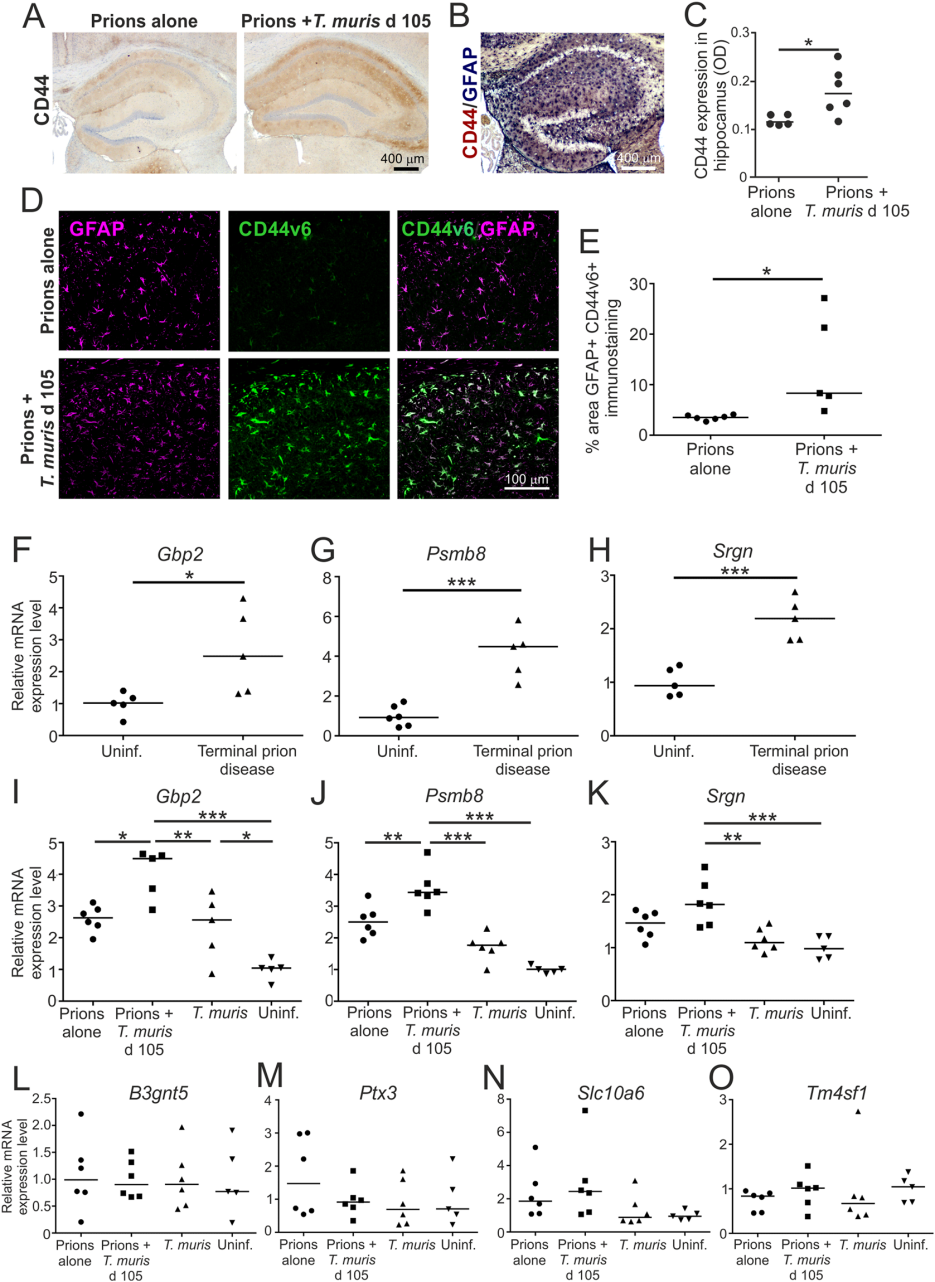
Earlier onset of astrocyte activation in the brains of mice co-infected a low dose of *T. muris* during the pre-clinical phase of prion disease. Mice were first injected with ME7 scrapie prions directly into the CNS by IC injection and 105 d later they were orally co-infected with a low dose of *T. muris* infective eggs (~20/mouse). Age matched uninfected mice, and mice infected with prions alone or *T. muris* alone were used as controls. Brains were collected during the pre-clinical phase of disease at 140 d after prion infection. (**A**) Immunohistochemical (IHC) analysis of CD44 expression in the hippocampus of mice from each group. Haematoxylin was used as a nuclear counterstain (blue). (**B**) IHC showed that CD44 (blue/back) was expressed in association with GFAP+ astrocytes (red/brown) in the brains of mice infected with prions. (**C**) Comparison of the magnitude of the CD44+ immunostaining (OD, relative optical density) in the hippocampus of mice infected with prions alone or co-infected with *T. muris* at 105 d after prion infection. *P < 0.05, Student’s *t*-test, *n* = 5–6/group; horizontal bar, median. (**D**) Immunofluorescence microscopy showed that CD44v6 (green) was highly expressed in association with GFAP+ astrocytes (magenta) in the brains of mice co-infected with *T. muris* at 105 d after prion infection. (**E**) The % area of GFAP+CD44v6+ co-immunostaining was significantly increased in the hippocampus of mice co-infected with *T. muris* at 105 d after prion infection when compared to mice infected with prions alone. *P < 0.05, Student’s *t*-test, *n* = 5–6/group; horizontal bar, median. (**F–H**) RT–qPCR analysis revealed a significant increase in expression of the A1 astrocyte-associated genes *Gbp2*, *Psmb8* and *Srgn* in the brains of mice with terminal prion disease when compared to uninfected controls. (**I–K**) RT–qPCR analysis revealed a significant increase in expression of the A1 astrocyte-associated genes *Gbp2*, *Psmb8* and *Srgn* in the brains of mice co-infected with *T. muris* at 105 d after prion infection. (**L–O**) RT–qPCR analysis suggested no upregulation of the A2 astrocyte-associated genes *B3gnt5*, *Ptx3*, *Slc10a6* and *Tm4sf1* in the brains of mice co-infected with *T. muris* at 105 d after prion infection when compared to uninfected controls. In panels F-O, gene expression data were normalized so that the relative mean expression level in uninfected mice was 1.0. *P < 0.05; **P < 0.01; ***P < 0.001; Student’s *t*-test, *n* = 5–6/group; horizontal bar, median.

The reactive astrocytes that are induced in the brain after various pathological insults can contribute to the neurodegeneration, or aid the recovery of the CNS, dependent upon their subclass. The A1 subclass of reactive astrocytes display neurotoxic properties and can induce neurodegeneration, whereas the A2 astrocytes are neurotrophic and can provide neuroprotection^21,29,30^. Expression of CD44 expression is significantly elevated in neurotoxic A1 astrocytes^21^. We have also shown that CD44, including the CD44v6 isoform, is expressed highly in specific astrocyte populations in brain areas associated with pathology during CNS prion disease^31^. Here, low levels of reactive astrocyte-associated CD44 expression were detected in the hippocampus at 140 d post infection with prions alone (Fig. 6A,B). However, CD44 expression was significantly higher in the brains of mice co-infected with *T. muris* at 105 d after prion infection (Fig. 6A,C). Significantly higher levels of reactive astrocyte-associated CD44v6 isoform expression were also detected in the hippocampus of the co-infected mice at this time when compared to mice infected with prions alone (Fig. 6D,E). Expression of mRNA encoding the neurotoxic A1 astrocyte markers^21^ guanylate binding protein 2 (*Gbp2*), proteasome subunit beta 8 (*Psmb8*) and serglycin (*Srgn*) was elevated in the brains of mice infected with prions alone at the terminal stage (Fig. 6F–H). At d 140 post infection with prions alone there was only modest increase in the expression of these A1 astrocyte-associated genes (Fig. 6I–K). However, on d 140 in the brains of mice co-infected with prions and *T. muris* the expression of *Gbp2*, *Psmb8* and *Srgn* was significantly higher when compared to uninfected mice and mice infected with prions alone (Fig. 6I–K), suggesting an earlier onset of expression of A1 astrocyte-associated genes in the brains of co-infected mice. In contrast, expression of the A2 astrocyte-associated genes *B3gnt5*, *Ptx3*, *Slc10a6* and *Tm4sf1* was not upregulated on d 140 in the brains of the co-infected mice above the level observed in uninfected mice (Fig. 6L–O). Together, these data suggest that co-infection with a low dose of *T. muris* during the pre-clinical phase of prion disease coincided with the earlier onset of expression of certain markers associated with neurotoxic A1 astrocyte activation.

### Reactive astrocytes express IFN-γ receptor 1 (IFNGR1) during CNS prion disease

**Figure 7:**
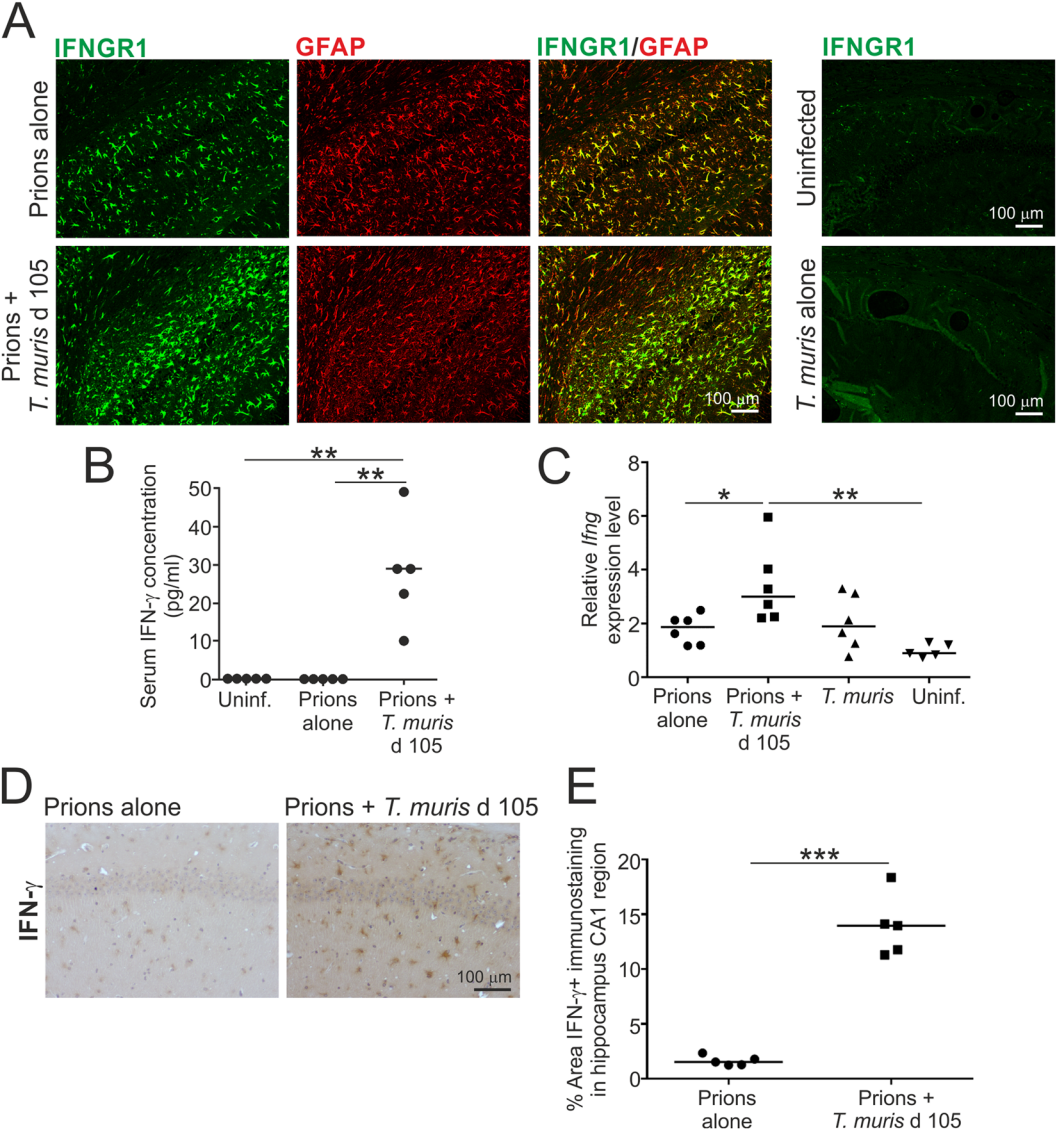
Astrocytes express high levels of IFN-γ receptor 1 (IFNGR1) in the brain during the pre-clinical phase of prion disease. Mice were first injected with ME7 scrapie prions directly into the CNS by IC injection and 105 d later they were orally co-infected with a low dose of *T. muris* infective eggs (~20/mouse). Age matched uninfected mice, and mice infected with prions alone or *T. muris* alone were used as controls. Brains were collected during the pre-clinical phase of disease at 140 d after prion infection. (**A**) Immunofluorescence microscopy shows that IFNGR1 (green) was highly expressed in association with GFAP+ astrocytes (red) in the brains of mice infected with prions alone, or mice co-infected with *T. muris* at 105 d after prion infection. (**B**) Detection of IFN-γ in the serum of mice co-infected with prions and *T. muris*. Each point represents data from an individual mouse. **P < 0.01; Student’s *t*-test, *n* = 5 mice/group; horizontal bar, median. (**C**) RT–qPCR analysis revealed a significant increase in *Ifng* expression in the brains of mice co-infected with *T. muris* at 105 d after prion infection. Each point represents data from an individual mouse. *P < 0.05; Student’s *t*-test, *n* = 5–6 mice/group; horizontal bar, median. (**D**) IHC suggested that IFN-γ was predominantly detected in association with astrocytes in the hippocampus of mice co-infected with *T. muris* at 105 d after prion infection. Haematoxylin was used as a nuclear counterstain (blue). (**E**) Comparison of the magnitude of the IFN-γ+ immunostaining in the CA1 region of the hippocampus of mice infected with prions alone or co-infected with *T. muris* at 105 d after prion infection. *P < 0.001, Student’s *t*-test, *n* = 5–6/group; horizontal bar, media*n*.

Limited, if any, astrocyte-associated IFNGR1 expression was detected in the brains of uninfected mice, or mice infected with *T. muris* alone (Fig. 7A). However, coincident with a Th1-polarised immune response induced by low dose *T. muris* infection, and the shorter prion disease survival times, the reactive GFAP+ astrocytes throughout the brains of mice that were co-infected with prions and *T. muris* also expressed IFNGR1 highly (Fig. 7A). This coincided with significantly elevated levels of IFN-γ in the sera of mice co-infected with prions and *T. muris* (Fig. 7B). Modest but significantly elevated levels of mRNA-encoding IFN-γ (*Ifng*) were also detected in the brains of mice co-infected with prions and *T. muris* (Fig. 7C), implying that certain cell populations within the CNS of the co-infected mice may also be producing this cytokine. Consistent with this hypothesis, IHC analysis detected IFN-γ predominantly in association with astrocytes in the brains of the co-infected mice (Fig. 7D,E). Although IFNGR1 was also expressed highly in reactive astrocytes in the brains mice infected with prions alone (Fig. 7A), IFN-γ expression was undetectable in the sera and brains of these mice (Fig. 7B–E) consistent with previous data^32^.

**Figure 8:**
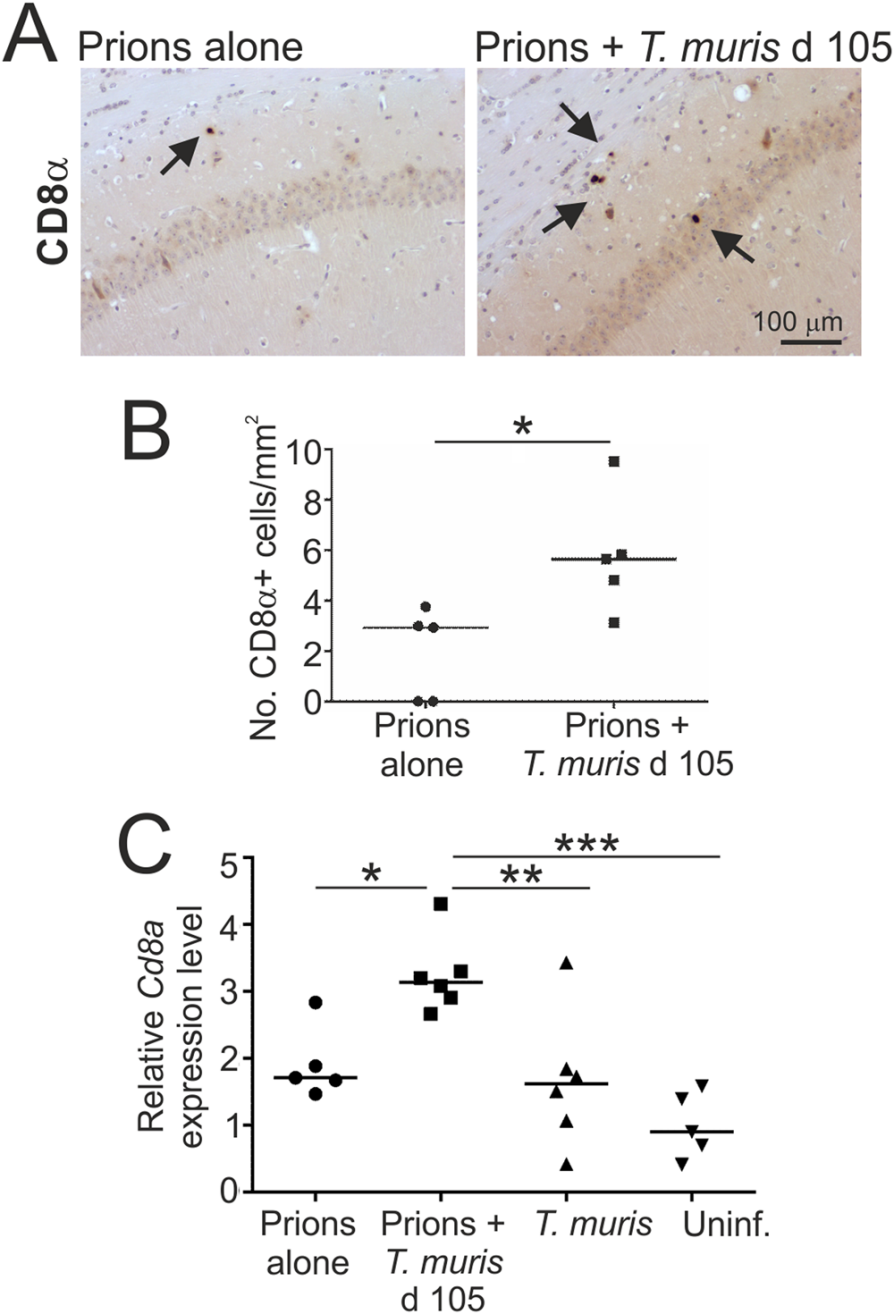
Detection of CD8α+ cells within the hippocampus during CNS prion disease. Mice were first injected with ME7 scrapie prions directly into the CNS by IC injection and 105 d later they were orally co-infected with a low dose of *T. muris* infective eggs (~20/mouse). Age matched uninfected mice, and mice infected with prions alone or *T. muris* alone were used as controls. Brains were collected during the pre-clinical phase of disease at 140 d after prion infection. (**A**) IHC analysis detected an increased abundance of CD8α+ cells (arrows) within the hippocampus of mice co-infected with *T. muris* at 105 d after prion infection. Haematoxylin was used as a nuclear counterstain (blue). (**B**) A significant increase in the abundance of CD8α+ cells was detected within the hippocampus of mice co-infected with *T. muris* at 105 d after prion infection. Data are presented as number of cells/mm^2^ hippocampus. Each point represents data from an individual mouse. **P < 0.01; Student’s *t*-test, *n* = 5–6 mice/group; horizontal bar, median. (**C**) RT–qPCR analysis revealed a significant increase in *Cd8a* expression in the brains of mice co-infected with *T. muris* at 105 d after prion infection. Each point represents data from an individual mouse. **P < 0.01; ***P < 0.001; Student’s *t*-test, *n* = 5–6 mice/group; horizon*t*al bar, median.

Chronic *T. muris* infection is known to stimulate the development of high levels of IFN-γ+ CD8+ T cells within the gut draining lymph nodes^33^, which can accumulate in other sites such as the bone marrow^34^. Low levels of CD8+ T cells have been reported to infiltrate the brains of mice and humans during CNS prion disease^35^. Here, few if any CD8α+ cells were detected by IHC in the hippocampus at 140 d post infection with prions alone, whereas a significantly increased abundance of CD8α+ cells was detected in the co-infected mice (Fig. 8A,B). This coincided with a significant increase in *Cd8a* (encoding CD8α) mRNA expression in brains from the co-infected mice (Fig. 8C). These data suggested that co-infection with *T. muris* may have stimulated the recruitment of CD8+ T cells into the prion disease-affected brain.

## Discussion

Systemic inflammation can have a significant impact on the progression of some neurodegenerative disorders^4–8,36^. Consistent with the suggestion that systemic inflammation may affect CNS prion disease pathogenesis^20^, we show that the onset of clinical prion disease was accelerated in mice co-infected with a low dose of *T. muris* that develop a chronic Th1-polarized systemic immune response. The reduced prion disease survival times in mice co-infected with a low dose of *T. muris* coincided with enhanced astrocyte activation during the preclinical phase, including the earlier onset of expression of certain neurotoxic A1 astrocytes-associated markers. When A1 astrocytes are induced they rapidly lose their homeostatic functions and instead gain neurotoxic properties that can kill neurons and oligodendrocytes^21^. This raises the hypothesis that the earlier onset of the reactive astrocyte activation in the brains of the co-infected mice may have contributed to the accelerated development of clinical prion disease, and suggests that pharmacological modulation of astrocyte phenotype may be beneficial during the CNS phase of prion disease.

Co-infection with a high dose of *T. muris* that induces a protective Th2 polarized immune response did not affect prion disease pathogenesis. Thus, our data show that the earlier onset of clinical signs of prion disease and reduced survival times were specific to mice with a chronic parasite-specific, IFN-γ-mediated, Th1 polarized immune response induced by co-infection with a low dose of *T. muris*^25,26^. The effects on prion disease pathogenesis observed in the current study are specific to the CNS phase of prion disease, as we have previously shown that co-infection with *T. muris* around the time of oral prion exposure does not affect prion disease pathogenesis^37^. Systemic production of the chemokine CCL5 was previously identified as the critical factor responsible for the augmented ischemic brain injury in mice undergoing a similar Th1 polarized immune response to *T. muris* infection^8^. However, we found no correlation between serum CCL5 concentrations and prion disease duration. Since prion disease pathogenesis and susceptibility are not affected in the absence of CCR5 (the receptor for CCL5)^38^, our data suggest that factors other than systemic CCL5 expression are responsible for the accelerated CNS prion disease pathogenesis in the co-infected mice.

We were unable to identify the precise molecular factor/s responsible for the effect of low dose *T. muris* infection on CNS prion disease pathogenesis. However, in support of the observation that this effect was specific to a chronic parasite-specific, IFN-γ-mediated Th1 polarized immune response^26^, we observed that reactive astrocytes expressed IFNGR1 highly during CNS prion disease. Stimulation of astrocytes with IFN-γ can induce neurotoxic activity^39^. This raises the hypothesis that IFN-γ-mediated stimulation may have induced the earlier onset of neurotoxic activity in reactive astrocytes in the co-infected mice during the pre-clinical phase of prion disease. An independent study has also reported that the expression of several IFN-γ inducible genes is upregulated in the brains of mice infected with prions^40^, and some of these genes are induced in astrocytes by IFN-γ stimulation^41^. Amongst these is the A1-astrocyte-associated gene *Gbp2* that we show is also upregulated in the brain during prion disease. Expression of proteosomal subunit genes including *PSMB8* is also inducible by IFN-γ stimulation^42^. Our previous pathway analysis also implicated IFN-γ as an upstream regulator during CNS prion disease pathogenesis^43^, and a transcriptomic signature enriched in IFN pathway-associated genes has been observed in A1 neurotoxic astrocytes in the aged human brain^44^. Expression of IFNGR1 mRNA is also specifically upregulated in reactive astrocytes in the hippocampus following entorhinal deafferentation^45^, and astrocyte-associated IFNGR expression has been detected in affected regions of the brains of patients with Alzheimer’s disease, amyotrophic lateral sclerosis and Parkinson’s disease^39^. Although IFN-γ was detected in the sera of mice co-infected with prions and *T. muris*, it was undetectable in mice infected with prions alone, consistent with previous data^32^. This implies that the upregulated expression of IFNGR1 on reactive astrocytes during CNS prion disease primes them to respond to subsequent IFN-γ-mediated stimulation.

The majority of astrocytes maintain direct physical contacts with vascular endothelial cells and pericytes via their endfeet, and these cells provide trophic support to astrocytes^46^. Thus, it is plausible that in the brains of mice co-infected with prions and *T. muris* the association of IFNGR1-expressing astrocytes with blood vessels would enable them to be directly stimulated by the elevated levels of IFN-γ in the serum. We also detected low levels of *Ifng* expression specifically in the brains of the co-infected mice, and our IHC analysis suggested IFN-γ was predominantly associated with astrocytes. This implies either that the astrocytes were producing this cytokine themselves in response to the parasite infection and being stimulated by it in an autocrine manner, or conversely that they had acquired IFN-γ from other sources.

Future studies using IFNGR1-deficient mice, and/or mice with conditional deficiencies in IFNGR1 or IFN-γ in astrocytes are clearly necessary to determine the influence that IFNGR1-mediated signalling has on astrocyte phenotype and CNS prion disease pathogenesis during the steady state, and in the presence of systemic inflammation. This will also help address whether systemically produced (serum) IFN-γ has the major influence on astrocyte phenotype, or whether astrocyte-derived IFN-γ modulates their phenotype in an autocrine manner.

Although low levels of CD4+ and CD8+ T cells have been reported to infiltrate the brains of mice and humans during CNS prion disease^35^, T cells do not contribute to CNS prion disease pathogenesis during the steady-state^47–49^. We detected a significantly increased abundance of CD8α+ cells in the hippocampus of the co-infected mice, suggesting that co-infection with *T. muris* stimulated the recruitment of CD8+ T cells into the prion disease-affected brain. Studies using an experimental reovirus-induced encephalitis model show that IFN-γ can induce changes in the endothelium of the blood-brain barrier that lead to increased permeability and the infiltration of CD3+ and CD8+ T cells into the CNS^50^. In the current study it is plausible that serum IFN-γ produced in response to low dose *T. muris* similarly affected the integrity of the blood-brain barrier in the of the co-infected mice, leading to increased infiltration of CD8+ T cells into the brain. The IFN-γ+ CD8+ T cells generated in response to low dose *T. muris* infection can accumulate in other sites such as the bone marrow^34^. This suggests that in addition to the increased number, the higher proportion of the CD8+ T cells found in the brains of co-infected mice may also be expressing IFN-γ. Clonally-expanded CD8+ T cells have been shown to enter the cerebrospinal fluid and brains of patients affected with Alzheimer’s disease^51^. Transcriptional analysis showed these cells had enhanced expression of genes encoding pro-inflammatory cytokines, cytotoxic granules and proteases, implying a potential role in the neurodegeneration. However, it remains to be determined whether CD8+ T cells are also sources of IFN-γ in the CNS, exhibit cytotoxic properties and contribute directly to the neurodegeneration in mice co-infected with prions and *T. muris*.

When the mice were co-infected with a low dose of *T. muris* on d 49 and d 77 this did not affect the onset of prion disease. The peak of the parasite-specific, IFN-γ-mediated, Th1-polarised inflammatory response in these instances was timed so that it would occur around d 84 and d 112 post injection with prions, and before the development of significant neuropathology (Fig. 1C,D). Data from the analysis of AKR mice that also develop a chronic Th1-polarised immune response after *T. muris* infection^52^ suggest that the magnitude of the systemic IFN-γ response in these mice is likely to have declined significantly by the time the co-infected mice began to display detectable clinical signs of prion disease (~152 days in current study). However, when the mice were co-infected on d 105, the peak of the IFN-γ response on d 140 was associated with the earlier activation of the astrocytes in the brain, and accelerated prion disease. Here, the timing of the *T. muris* co-infection was selected so that the peak of the inflammatory response coincided with the presence of detectable signs of neurodegeneration (spongiform pathology/vacuolation) in the CNS at d 140 (Fig. 1D), and just before the onset of clinical signs. This suggests that the presence of a strong systemic IFN-γ-mediated, Th1-polarized inflammatory response may accelerate CNS prion disease in mice with detectable signs of neurodegeneration. Conversely, a strong systemic Th1-polarized inflammatory response is unlikely to influence the onset of CNS prion disease if it occurs much earlier during the infection and before the development of the neuropathology.

Widespread dietary exposure of the UK human population to bovine spongiform encephalopathy (BSE) prions is likely to have occurred during the epidemic in the UK cattle herd in the 1980s^53,54^. Fortunately however, relatively few human cases of variant Creutzfeldt-Jakob disease (vCJD) due to the consumption of BSE contaminated food^55^ have been recorded in the UK (178 definite and probable cases, January 2020)^56^. However, histopathological analyses of PrP^d^ accumulation in archived human appendix and tonsil samples have proposed that the incidence of individuals with pre-clinical vCJD infection could be higher^57,58^. This discrepancy suggests the potential existence of subclinical carriers. Our data show that CNS prion disease was accelerated in mice co-infected with a low dose of *T. muris* during the pre-clinical phase. Whether inflammatory mediators produced in response to pathogen co-infections similarly accelerate the progression of CNS prion disease in pre-clinically-affected individuals remains to be determined.

Our study addresses an important gap in our understanding of how systemic inflammation in response to co-infection with a gastrointestinal pathogen may accelerate CNS prion disease pathogenesis in pre-clinically-affected individuals. Expression of IFNGR may also be upregulated in the brains of patients with Alzheimer’s disease, amyotrophic lateral sclerosis, Parkinson’s disease and multiple sclerosis^39^. Thus, our data imply that targeting of the IFN-γ response in the immune system and/or the downstream response in astrocytes may have therapeutic value in delaying neurodegenerative disease progression across a range of disorders.

## Materials and Methods

### Ethics statement

Ethical approvals for the *in vivo* mouse experiments were obtained from The Roslin Institute’s and University of Edinburgh’s ethics committees. These experiments were also performed under the authority of UK Home Office Project Licences (PPL60/4325 & PA75389E7) in accordance within the guidelines and regulations of the UK Home Office ‘Animals (scientific procedures) Act 1986’. Appropriate care was provided to minimise harm and suffering, and anaesthesia was administered where necessary. Mice were humanely culled at the end of the experiments by cervical dislocation.

### Mice

This study used six to eight weeks old female C57BL/6J mice (Charles River, Margate, UK), and these were maintained under SPF conditions.

### Prion infection

Mice were injected IC with 20 µl of a 1% (v/w) terminal scrapie brain homogenate, containing approximately 1 × 10^4^ IC ID_50_ units of ME7 scrapie prions. The mice were then coded and assessed blindly by independent technicians at weekly intervals until they displayed definite clinical signs of prion disease, from which time they were assessed at daily intervals and daily culled as described^59^. The early clinical signs of prion disease in mice infected with ME7 scrapie prion can include weight loss, starry coat, hunched posture and jumpy behaviour. During the later stages of prion disease the clinical signs may also include hind-limb ataxia, limited movement and decreased awareness, upright tail, wet genitals, discharge from eyes and/or blinking eyes. Following assessment for the above clinical signs of prion disease the mice were scored as “unaffected,” “possibly affected,” and “definitely affected”. Mice were culled at the clinical endpoint of disease. This was defined as either after two consecutive “definite” ratings, or after a third “definite” rating within four consecutive weeks. Survival times were recorded as the duration from day of IC injection with prions to day of cull at the clinical endpoint. Histopathological assessment of the magnitude and distribution of the prion disease-specific spongiform pathology in the brain was used to confirm the clinical status of each mouse as described^28^. All the mice presented in Table 1 were injected IC with prions on the same day using inoculum derived from the same pool of terminal scrapie brain homogenate.

### Trichuris muris infection

*T. muris* was maintained as described^24^. Mice were infected orally by gavage with either ~20 (low dose) or ~200 (high dose) infective eggs and worm burdens in the large intestine were determined as described^60,61^. Mean worm burdens at the peak of inflammation following infection with ~20 infective eggs (35 d) or ~200 infective eggs (21 d) were 2 ± 1 and 20 ± 7, respectively (*n* = 3–4/group).

### *T. muris* Antigen-specific serum antibody responses

Serum *T. muris* excretory/secretory (E/S) antigen-specific IgG1 and IgG2c concentrations were measured by ELISA as described^27,62,63^.

### Chemokine and cytokine quantitation

Serum CCL5 concentrations were determined by using either a mouse/rat CCL5/RANTES Quantikine ELISA kit (Bio-techne, Abingdon, UK) or a mouse RANTES FlowCytomix Simplex kit (Ebioscience, Paisley, UK), according to the manufacturers’ instructions. Serum IFN-γ concentrations were determined using a MILLIPLEX MAP mouse TH17 magnetic bead panel (Merck Millipore, Watford, UK) array according to the manufacturer’s instructions.

### Immunohistochemistry

Brains were fixed in periodate-lysine-paraformaldehyde and then embedded in paraffin wax. To enhance the detection of disease-specific PrP (PrP^d^) sections (5 µm) were first treated with hydrated autoclaving (15 min, 121 °C, hydration) followed by immersion in formic acid (98%) for 5 min. Sections were then immunostained with 1B3 PrP-specific polyclonal antiserum^64^. For all other immunostainings the sections were autoclaved as above in Target Retrieval Solution (DAKO, Ely, UK). GFAP was detected using polyclonal rabbit anti-glial fibrillary acidic protein (DAKO). N-terminal (pan-) CD44 was detected using biotinylated anti-CD44 (clone IM7; Biolegend, London, UK). The CD44 variant 6 isoform was detected using rat anti-mouse CD44v6 (Clone 9A4; eBioscience, Hatfield, UK). CD8+ cells were detected using anti-CD8α (D4W2Z) XP rabbit monoclonal antibody (mAb) (Cell signalling Technology, London, UK). IFN-γ was detected using anti-IFN-γ mAb (clone XMG1.2; ThermoFisher Scientific, Perth, UK). INFGR1 was detected using biotinylated anti-CD119 (IFN-γ R α chain; clone 2E2; Biolegend). AIF-1+ (also known as Iba1) microglia were detected using polyclonal anti-rabbit Iba1 antibody (Wako Chemicals GmbH, Neuss, Germany). For light microscopy, appropriate species-specific biotin-conjugated secondary antibodies (Jackson ImmunoResearch Europe Ltd., Ely, UK) were then applied followed by HRP-conjugated to the avidin-biotin complex (ABC kit, Vector Laboratories, Peterborough, UK). For anti-CD44 and anti-GFAP co-immunostaining, CD44 was detected using the HRP substrate Vector NovaRed (Vector Laboratories) and GFAP detected with alkaline phosphatase conjugated anti-rabbit antibody (Jackson Immunoresearch) and 5-bromo-4-chloro-3-indolyl phosphate/nitro blue tetrazolium (BCIP/NBT) substrate (Merck KGaA, Darmstadt, Germany). Positive immunolabelling was detected using diaminobenzidine (DAB; Sigma). Haematoxylin was used a nuclear counterstain. For immunofluorescence analysis, GFAP immunostaining was visualised using goat-anti rabbit iFluora594 (AAT Bioquest, Sunnyvale, USA) and CD44v6 immunostaining was detected using biotin-SP AffiniPure Goat anti-rat IgG (H + L; Jackson Immunoresearch). IFNGR1 and CD44v6 immunostaining were then visualised using an ABC-HRP kitd (Vector Laboratories) and the HRP substrate Tyramide iFluora488 (AAT Bioquest). Sections were mounted in fluorescence mounting medium (DAKO) before analysis, and images captured using a confocal laser scanning LSM710 microscope with ZEN pro software (Zeiss; https://www.zeiss.com/microscopy/int/products/microscope-software/zen.html). Sections were stained with haematoxylin and eosin for histopathological assessment of the spongiform pathology^28^.

### Image analysis

Images were analysed using ImageJ software^65^ (http://imagej.nih.gov/ij) on coded sections blinded to the operator as described previously^66,67^. Data are presented as the percentage of positively stained pixels for each cell marker in the region of interest. The magnitude of the CD44 immunostaining on DAB-stained sections was compared as previously described^31^. Briefly, the optical density (OD) values for CD44 immunostaining were calculated using ImageJ software following H-DAB deconvolution. Mean grey OD values were measured from DAB grayscale images (scaled 0–255) and converted using the formula OD = log_10_ (255/mean grey value). Images were typically captured from 5–6 mice/group, and the figure legends provide the sample sizes for each parameter analysed.

### Immunoblot analysis

Homogenates of frozen half brains (10% weight/volume) were prepared in NP-40 lysis buffer (1% NP-40, 0.5% sodium deoxycholate, 150 mM NaCl, 50 mM Tris-HCl [pH 7.5]). Samples were then treated in the presence/absence of 20 µg/ml proteinase K for 1 h at 37 °C. Following electrophoresis and electroblotting onto polyvinylidene difluoride membranes. PrP was detected using mAb 7A12^68^ as described previously^69^. Supplementary Fig. S1 shows the uncropped immunoblot presented in Fig. 5F.

### RT-qPCR

Half brains were snap-frozen at the temperature of liquid nitrogen, homogenized using Lysing Matrix D tubes (MP Biomedicals, Cambridge, UK) and a Ribolyser tissue homogenizer (Bio-Rad Laboratories, Watford, UK). Total RNA was extracted using RNABee (AmsBio, Abingdon, UK) and purified using an RNeasy Mini kit (Qiagen, Manchester, UK). Genomic DNA was then removed by treatment with RNase-free DNase I (Promega, Southampton, UK). First strand cDNA synthesis was performed using 5 µg total RNA and the SuperScript III First-Strand Synthesis System for RT-PCR (Life Technologies, Waltham, MA, USA). RT-qPCR was then performed using the primers listed in Table 2 and FastStart Universal SYBR Green Master mix (Rox; Sigma-Aldrich, Poole, Dorset, UK) on a MX3005P RT-qPCR system (Agilent Technologies LDA UK Ltd, Stockport, Cheshire, UK). Cycle threshold values were analysed using MxPro software (Agilent Technologies LDA UK Ltd; https://www.agilent.com/en/product/real-time-pcr-(qpcr)/real-time-pcr-(qpcr)-instruments/mx3000-mx3005p-real-time-pcr-system-software/mxpro-qpcr-software-232751), normalized relative to the reference gene *Rpl19* using the ΔΔCT method and expressed as relative gene expression values compared to the uninfected control group.

**Table 2:** Primers used for RT-qPCR analysis.

Gene	Forward primer	Reverse primer
*B3gnt5*	CGTGGGGCAATGAGAACTAT	CCCAGCTGAACTGAAGAAGG
*Cd8a*	CAGAGACCAGAAGATTGTCG	TGATCAAGGACAGCAGAAGG
*Gbp2*	GGGGTCACTGTCTGACCACT	GGGAAACCTGGGATGAGATT
*Ifng*	TGAGCTCATTGAATGCTTGG	ACAGCAAGGCGAAAAAGGAT
*Psmb8*	CAGTCCTGAAGAGGCCTACG	CACTTTCACCCAACCGTCTT
*Ptx3*	AACAAGCTCTGTTGCCCATT	TCCCAAATGGAACATTGGAT
*Slc10a6*	GCTTCGGTGGTATGATGCTT	CCACAGGCTTTTCTGGTGAT
*Srgn*	GCAAGGTTATCCTGCTCGGA	TGGGAGGGCCGATGTTATTG
*Tm4sf1*	GCCCAAGCATATTGTGGAGT	AGGGTAGGATGTGGCACAAG
*Rpl19*	GAAGGTCAAAGGGAATGTGTTCA	CCTTGTCTGCCTTCAGCTTGT

### Statistical analyses

Data are presented as mean ± SEM. Unless indicated otherwise, significant differences between samples in different groups were determined by Student’s *t*-test. In instances where there was evidence of non-normality, a Mann-Whitney *U* test was used. Values of *P* < 0.05 were accepted as significant.

## Supplementary information

Supplementary material.

## Data Availability

All relevant data are presented within the paper.
