# Supplementary material for: Accelerated onset of CNS prion disease in mice co-infected with a gastrointestinal helminth pathogen during the preclinical phase
Source: Sci Rep. 2020 Mar 12;10:4554. doi: 10.1038/s41598-020-61483-4 (PMC7067812; doi:10.1038/s41598-020-61483-4)

Accelerated onset of CNS prion disease in mice co-infected with a gastrointestinal helminth pathogen during the preclinical phase

David S. Donaldson, Barry M. Bradford, Kathryn J. Else & Neil A. Mabbott

Supplementary Information

Supplementary Figure S1. Image of the uncropped immunoblot used in Fig. 5F.

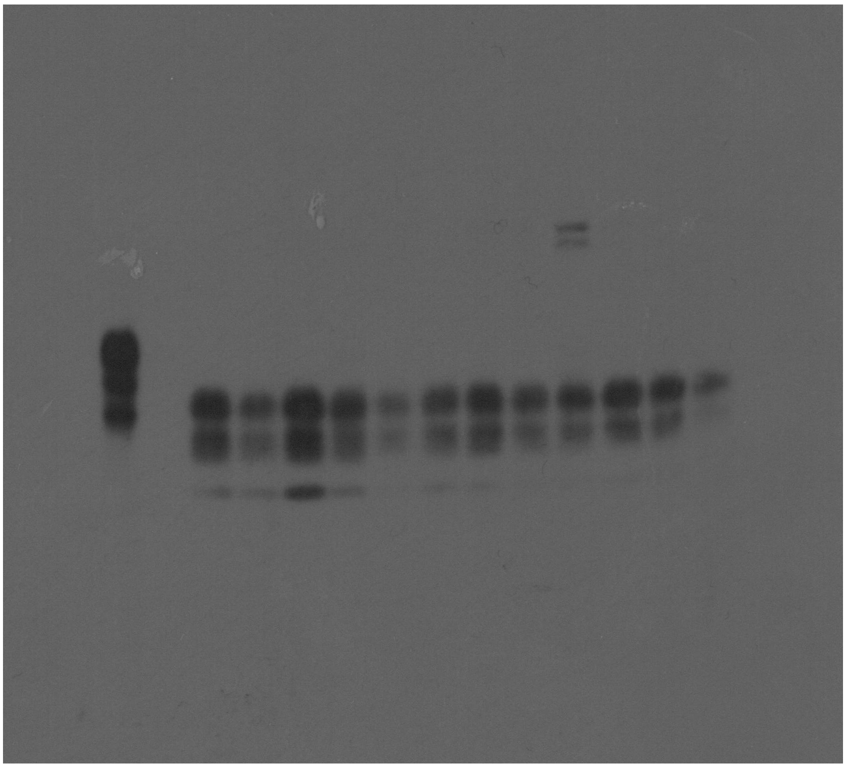

Supplement: Supplementary file 1 — Supplementary material. [file 41598_2020_61483_MOESM1_ESM.pdf]
